# Lipid Peroxidative Damage on Cisplatin Exposure and Alterations in Antioxidant Defense System in Rat Kidneys: A Possible Protective Effect of Selenium

**DOI:** 10.3390/ijms13021790

**Published:** 2012-02-08

**Authors:** Branka I. Ognjanović, Nataša Z. Djordjević, Miloš M. Matić, Jasmina M. Obradović, Jelena M. Mladenović, Andraš Š. Štajn, Zorica S. Saičić

**Affiliations:** 1 Institute of Biology and Ecology, Faculty of Science, University of Kragujevac, Radoja Domanovića 12, 34000 Kragujevac, Serbia; E-Mails: natasadj@kg.ac.rs (N.Z.D.); milosmatic@kg.ac.rs (M.M.M.); jasmina.m.obradovic@gmail.com (J.M.O.); jelena.m.mlad@gmail.com (J.M.M.); astajn@kg.ac.rs (A.Š.Š.); 2 Department of Physiology, Institute for Biological Research “Siniša Stanković”, University of Belgrade, Bulevar despota Stefana 142, 11000 Belgrade, Serbia; E-Mail: zorica.saicic@ibiss.bg.ac.rs

**Keywords:** cisplatin, selenium, nephrotoxicity, lipid peroxidation, antioxidant defense system

## Abstract

Cisplatin (*Cis*-diamminedichloroplatinum II, CP) is an important chemotherapeutic agent, useful in the treatment of several cancers, but with several side effects such as nephrotoxicity. The present study investigated the possible protective effect of selenium (Se) against CP-induced oxidative stress in the rat kidneys. Male Wistar albino rats were injected with a single dose of cisplatin (7 mg CP/kg b.m., i.p.) and selenium (6 mg Se/kg b.m, as Na_2_SeO_3_, i.p.), alone or in combination. The obtained results showed that CP increased lipid peroxidation (LPO) and decreased reduced glutathione (GSH) concentrations, suggesting the CP-induced oxidative stress, while Se treatment reversed this change to control values. Acute intoxication of rats with CP was followed by statistically significant decreased activity of antioxidant defense enzymes: superoxide dismutase (SOD), catalase (CAT), glutathione peroxidase (GSH-Px), glutathione reductase (GR) and glutathione-*S*-transferase (GST). Treatment with Se reversed CP-induced alterations of antioxidant defense enzyme activities and significantly prevented the CP-induced kidney damage.

## 1. Introduction

Cisplatin (*cis*-diammine-dichloroplatinum II, CP) has been considered one of the most effective chemotherapeutic agents, utilized for treatment of a variety of human solid tumors. Activity has been demonstrated against a variety of tumors, particularly of head and neck, ovarian, testicular, esophageal, bladder and small cell lung cancers [1]. The clinical use of CP is often limited due to its undesirable side effects, nephrotoxicity and neurotoxicity being the most severe and dose-limiting ones [2]. CP-induced kidney damage is associated with increased kidney vascular resistance and histological damage to proximal tubular cells [1,3,4]. The cytotoxicity of CP is considered to be due to a combination of factors, including peroxidation of the cell membrane, mitochondrial dysfunction, inhibition of protein synthesis, and DNA injury [3,5,6]. The most common adverse effect limiting the use of CP is nephrotoxicity that develops primarily in the S3 segment of the proximal tubule [7]. Although reactive oxygen species (ROS) have been considered to play a central role in this injury, the exact roles of free radicals and the mechanisms underlying the beneficial effects of free radical scavengers have not been fully evaluated [1–3,8,9].

The pathogenesis of kidney damage caused by CP is generally considered oxidative damage [1,2]. Administration of CP causes an increase in lipid peroxide levels and a decrease in the activity of antioxidant defense enzymes that prevent or protect from lipid peroxidation in the tissues. Cisplatin accumulates in the tubular epithelial cells of proximal kidney tubule, causing nephrotoxicity, characterized by morphological destruction of intracellular organelles, cellular necrosis, loss of microvilli, alterations in the number and size of the lysosomes and mitochondrial vacuolization, followed by functional alterations including inhibition of protein synthesis, GSH depletion, lipid peroxidation and mitochondrial damage [1,2,4,8–10].

Recent studies have focused on the role of antoxidants in CP toxicity. The administration of antioxidants and other agents have been shown to ameliorate CP-induced toxicity in various species of animals [7,11–18].

Selenium (Se) is an essential trace element which plays an important role in a number of biological processes in humans and many other forms of life. Many experimental studies of animals have demonstrated that the deficiency of Se induces some pathological conditions (coronary heart disease, liver necrosis) and is an important risk factor in the etiologies of these diseases [19–23]. Biological and medical advances in the area of Se provide interest in Se for both its antioxidant properties through seleno-enzyme incorporation and its direct pro-oxidant toxic effect through seleno-compounds [21,22]. Se is an essential component of several enzymes such as glutathione peroxidase (GSH-Px), thioredoxin reductase (TR) and selenoprotein P (SeP), which contains Se as selenocysteine. It is also well known that Se is essential for cell culture when a serum-free medium is used [21–25].

Recent studies showed protective effects of Se against cadmium (Cd)-induced oxidative stress [26,27]. It has also been demonstrated that the chronic exposure to low levels of Cd and other toxic elements abolishes the cancer-protective effect of Se. An important property of Se is its interaction with other elements that may be present in food, water, at the workplace and in the environment. Se functions as an antagonist to the toxicity of metals such as Hg, Cd, As, Ag, Pb and Cu [28,29]. The arsenic-, platinum- and gold-containing drugs significantly influence the fate of exogenous Se, whereby they may adversely affect the availability of this element, essential for synthesis of selenoenzymes [30].

The aim of the present study was to investigate a protective effect of Se pretreatment on lipid peroxidation (LPO) and reduced glutathione (GSH) concentrations and activity of antioxidant defense enzymes: superoxide dismutase (SOD), catalase (CAT), glutathione peroxidase (GSH-Px), glutathione reductase (GR), as well as glutathione-*S*-transferase (GST) in the kidney of rats, acutely treated with CP.

## 2. Experimental Methods

### 2.1. Chemicals

Cisplatin (*cis*-dichlorodiammine-platinum II, CP) and sodium selenite (Na_2_SeO_3_) were purchased from Merck Chemical Inc. (Darmstandt, Germany). Nicotinamide adenine dinucleotide phosphate (reduced form; NADPH); reduced (GSH) and oxidized (GSSG) glutathione; 1-chloro-2,4-dinitrobenzene (CDNB); *tert*-butyl hydroperoxide (*t* BOOH); glutathione reductase; DTNB (5,5′-dithio-bis-2-nitrobenzoic acid) and bovine serum albumin were purchased from Sigma Chemical Co. (St. Louis, MO, U.S.A). All other chemicals and reagents were of the highest commercially available purity.

### 2.2. Animals and Treatments

Male Wistar albino rats (about 3 months old, weighing 210–250 g) were used. The animals were kept under standard laboratory conditions (12 h light, 12 h dark and 21 ± 2 °C). All rats were housed in individual cages and given standard diet and tap water ad libitum. The University Committee of the Ethics of Animal Experimentation approved all animal experiments. The animals were divided into four groups (*n* = 6 per group) and treated as follows:

Group 1: Control rats, treated intraperitoneally (i.p.) with isotonic saline.
Group 2: Cisplatin (CP) (received i.p. a single dose of 7 mg CP/kg b.w.).
Group 3: Selenium (Se) (received a single i.p. injection of Na_2_SeO_3_ in the dose of 6 mg Se/kg b.w.).
Group 4: Se + CP (treated by Se 1 h before CP injection in the above mentioned amounts).

After the treatment (3 days after CP injection) all animals were sacrificed by decapitation. The kidney tissues were quickly excised, rinsed in ice-cold saline and used immediately or stored frozen at −80 °C until further biochemical analysis.

### 2.3. Tissue Preparation

The kidney tissues were minced and homogenized with a Thomas Sci Co. glass homogenizer (Teflon pestle) at 0–4 °C (10% w/v) using 0.25 M sucrose, 1 mM EDTA and 0.05 M Tris-HCl solution and pH 7.4. The homogenates were centrifuged (90 min at 10,000× *g*, 4 °C) and the supernatant was used for antioxidant defense enzyme activity assays and for total protein determination.

### 2.4. Biochemical Analysis

#### 2.4.1. Lipid Peroxidation Assay

Lipid peroxidation (LPO) was evaluated by measuring the MDA concentration. The tissues homogenates (using 1.15% KCl) were precipitated with trichloracetic acid. After centrifugation (1500× *g*, 15 min), the supernatant was mixed with TBA reagent (0.6%) and the mixture was kept at 100 °C for 1 h. The fluorescent reaction product was extracted with *n*-butanol and the fluorescence was measured in the organic phase, using a fluorescence spectrophotometer (excitation: 535 nm and emission: 555 nm) [31]. These results were expressed in nmol MDA/g tissue using a molar extinction coefficient for MDA of 1.56 × 10^5^ M^−1^·cm^−1^.

#### 2.4.2. Determination of Antioxidant Enzyme Activity

Superoxide dismutase (SOD, EC 1.15.1.1) activity was assayed in the supernatant by the epinephrine method [32]. The method is based on the measurement of the rate of epinephrine auto-oxidation inhibition by SOD contained in the examined samples in 50 mM sodium carbonate buffer pH 10.2, within the linear range of auto-oxidative curve. SOD activity was expressed as units/mg protein.

Catalase (CAT, EC 1.11.1.6) activity was measured by the method of Beutler [33]. The method is based on the rate of H_2_O_2_ degradation by the action of CAT contained in the examined samples and followed spectrophotometrically at 230 nm in 5 mM EDTA, 1 M Tris-HCl solution, pH 8.0. The enzyme activity was expressed in μmol H_2_O_2_ min/mg protein.

The activity of glutathione peroxidase (GSH-Px, EC 1.11.1.9) was determined using *t*-butyl hydroperoxide as a substrate by the method of Tamura *et al.* [34] and the activity was expressed as nmol NADPH oxidized/min/mg protein.

Glutathione reductase (GR, EC 1.6.4.2) activity was assayed by the method of Glatzle *et al.* [35] by measuring NADPH oxidation in the presence of oxidized glutathione and the activity was expressed as nmol NADPH oxidized/min/mg protein.

For determination of glutathione-*S*-transferase (GST, EC 2.5.1.18) activity, 1-chloro-2,4-dinitro benzene (CDNB) was used as a substrate [36] and the activity was expressed as nmol GSH used min/mg protein.

#### 2.4.3. Reduced Glutathione (GSH) Assay

The concentration of reduced glutathione (GSH) was measured by the method of Beutler [37]. Tissue samples for GSH assay were homogenized on ice with 20 volumes of precipitating solution (1.5 mL 100 mmol/L Na-phosphate/5 mmol/L EDTA buffer, pH 8.0 and 0.4 mL 25% metaphosphoric acid) and then centrifuged at 10,000 *g* for 30 min at 4 °C. The reaction mixture contained 0.5 mL of supernatant, 0.75 mL of Na-phosphate puffer (0.2 M, pH 7.4), 0.1 mL DTNB (5,5′-dithio-bis-2-nitrobenzoic acid) and 0.04 mL NaOH. The solution was kept at room temperature for 15 min and then read at 412 nm on a spectrophotometer. The concentration of GSH was expressed in μmol GSH/g protein.

#### 2.4.4. Protein Concentration Assay

The concentration of total proteins was determined by the biuret method [38] using Folin’s reagent and bovine serum albumin (BSA) as standard.

### 2.5. Statistical Analysis

All data are presented as means ± SD. Statistical significance of the results was evaluated by using one-way ANOVA (analysis of variance) test and post-comparison was carried out with Student’s *t*-test. A probability value less than 0.05 (*p* < 0.05) was considered statistically significant.

## 3. Results

The data presented in Table 1 show significant changes in the concentrations of LPO and GSH during the treatment of rats with CP and Se alone or in combination. The results showed that LPO concentration significantly increased in kidneys of rats treated with CP (by about 27%) (*p* < 0.05) in comparison to control. Pretreatment with Se was very effective in the prevention of oxidative damage induced by CP, which resulted in significantly lower LPO concentration. Alone Se treatment had showed no significant effect. Exposure to CP caused significant decrease of GSH concentration by 25.6% (*p* < 0.05) in kidney of rats, while Se treatment reversed this change to control values. No significant change in GSH was found in rats treated with Se + CP and Se only in comparison with control group.

**Figure 1:**
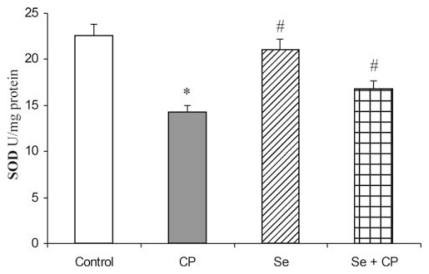
Effect of Se treatment on superoxide dismutase (SOD) activity in kidney of CP-treated rats. Values are expressed as means ± SD; *n* = 6 for each treatment group; CP: Cisplatin; Se: selenium; * *p* < 0.05 compared with control group, # *p* < 0.05 compared with CP group.

**Figure 2:**
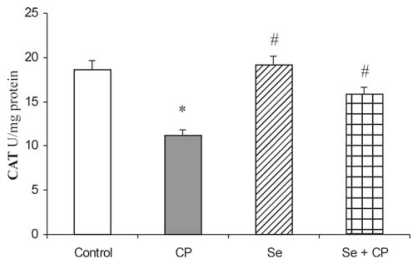
Effect of Se treatment on catalase (CAT) activity in kidney of CP-treated rats. Values are expressed as means ± SD; *n* = 6 for each treatment group; CP: Cisplatin; Se: selenium; * *p* < 0.05 compared with control group, # *p* < 0.05 compared with CP group.

**Figure 3:**
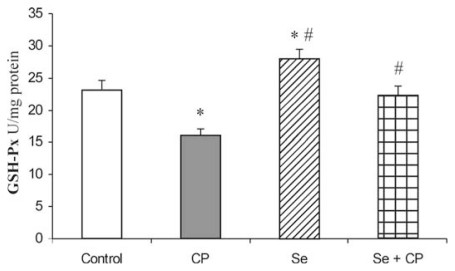
Effect of Se treatment on glutathione peroxidase (GSH-Px) activity in kidney of CP-treated rats. Values are expressed as means ± SD; *n* = 6 for each treatment group; CP :Cisplatin; Se: selenium; * *p* < 0.05 compared with control group, # *p* < 0.05 compared with CP group.

**Figure 4:**
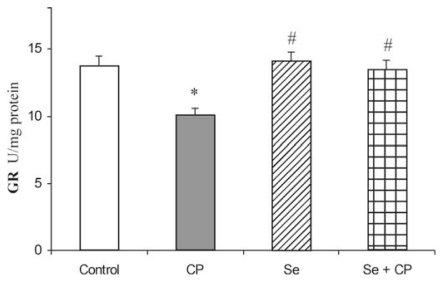
Effect of Se treatment on glutathione reductase (GR) activity in kidney of CP-treated rats. Values are expressed as means ± SD; *n* = 6 for each treatment group; CP: Cisplatin; Se: selenium; * *p* < 0.05 compared with control group, # *p* < 0.05 compared with CP group.

**Figure 5:**
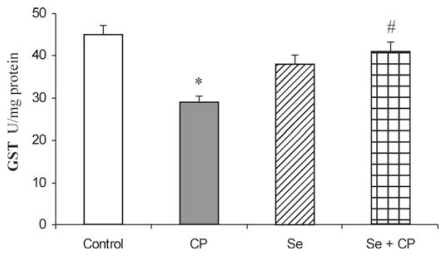
Effect of Se treatment on glutathione-*S*-transferase (GST) activity in kidney of CP-treated rats. Values are expressed as means ± SD; *n* = 6 for each treatment group; CP: Cisplatin; Se: selenium; * *p* < 0.05 compared with control group, # *p* < 0.05 compared with CP group.

Acute intoxication of rats with CP was followed by statistically significant decreased activities of all examined antioxidant defense enzymes (SOD, CAT, GSH-Px, GR and GST), (Figures 1–5). As represented in Figures 1 and 2, the exposure to CP caused the decrease of SOD and CAT activities (by about 37%) (*p* < 0.05) in kidney. In rats receiving Se only, and in rats pretreated with Se, the activities of SOD and CAT in kidney were similar to control values, but significantly increased (by about 21% and 26%) (*p* < 0.05) in comparison to the animals which received CP only.

The exposure to CP caused a significant decrease of GSH-Px (by 32.1%), GR (by 23.2%) and GST (by 36.8%) activities in comparison to control group (*p* < 0.05), (Figures 3–5). The protective role of Se in acute CP intoxication resulted in increased activities of GSH-Px (by about 25%) (Figure 3), GR (by about 15%) (Figure 4) and GST (by about 31%) (Figure 5) when compared to the animals given CP alone. The presence of the antioxidants minimized the toxic effects of CP on the affected enzymes. Treatment with Se alone significantly increased GSH-Px activity in kidney in comparison to the control (by about 16%) and CP-treated groups (by about 48%). Administration of Se alone and/or in combination with CP did not cause significant changes in activity of GR and GST enzymes in comparison with control group.

## 4. Discussion

Cisplatin (CP), a heavy metal complex is one of the most active drugs used in the treatment of a variety of cancers. However, the clinical usefulness of this drug is limited due to the development of nephrotoxicity as a side effect that may be produced in various animal models [2,16,18]. The treatment of tumor cells with CP provokes several responses including membrane peroxidation, dysfunction of mitochondria, inhibition of protein synthesis and DNA damage [1,4–6,8–10]. The current study demonstrates that Se provides protection against CP-induced nephrotoxicity in rats.

The data obtained in our study confirm that acute intoxication with CP causes a significant increase of LPO concentration in the kidney of rats (Table 1). CP-induced free radical production and LPO in tubular cells have been suggested to be responsible for the oxidative renal damage [1,3,8,14,39]. CP affects renal tissues where generated free radicals can interact with membrane lipids to produce their peroxidation, affecting cellular structure and function [2,17,18]. Kim *et al.* [12] observed that CP could promote the increase in lipid peroxidation *in vitro*. Pretreatment with Se was very effective in the prevention of oxidative damage induced by CP, which resulted in significantly lower LPO concentration in kidney. These results can be explained by the important role of Se in preventing lipid peroxidation and in protection of integrity and functioning of tissues and cells [13,20–23,26].

Therapeutic effects of CP are based on the interaction with DNA in the cell, preventing proliferation, and inducing apoptosis in tumor cells. It was also observed that CP treatment increased BUN (blood urea nitrogen) and creatinine levels. A marked increase in BUN and creatinine in serum and histopathological changes including vacuolation, necrosis, and protein casts were observed in proximal renal tubules on the second day after CP injection in rats [3,5,6,39]. Mukhopadhyay *et al.* [40] observed that cisplatin-induced mitochondrial ROS generation triggered inflammatory response, cell death, and kidney dysfunction/nephropathy. Cisplatin initially triggers oxidative stress in the mitochondria of kidney proximal tubular and endothelial cells, which is followed by a secondary wave of ROS/RNS (reactive nitrogen species) generation, deterioration of mitochondrial structure and function, an intense inflammatory response, histopathological injury and diminished renal function. Inflammation may further amplify oxidative/nitrative stress, and these interrelated processes eventually culminate in more concerted renal tubular and endothelial cell demise (both apoptotic and necrotic), secondary hypoxia, kidney dysfunction, and failure [40]. A single systemic dose of mitochondrially targeted antioxidants, MitoQ or Mito-CP, dose-dependently, prevented cisplatin-induced renal dysfunction. Mito-CP also prevented mitochondrial injury and dysfunction, renal inflammation, and tubular injury and apoptosis [40]. Recent studies showed protective effects of CoQ_10_ and Vit E against Cd-induced oxidative stress [41].

A large number of natural products and dietary components have been evaluated as potential chemoprotective agents. Many experimental studies in animals have demonstrated the ability of Se to prevent carcinogenesis [23]. Supplementation of the antioxidant vitamin C, E, curcumin, Se and other dietary components has been reported to inhibit lipid peroxide in various conditions such as CP-induced nephrotoxicity and hepatotoxicity [12–18,39].

In various studies that investigated the role of oxidant stress in the kidney, lipid peroxides are reported to be increased [13,14,16,18,39]. Enzymatic and non enzymatic antioxidant defense systems are present in the cell to prevent the integrity of biological membranes from oxidative processes caused by free radicals [42,43].

The kidney antioxidant defense system, such as SOD, CAT, GSH-Px, GR, GST activities (Figures 1–5), and reduced GSH concentration (Table 1) significantly decreased in the CP alone treated group of animals compared to the control. CP generates ROS such as superoxide anion (O_2_^−^) and hidroxil radicals (OH), and stimulates kidney lipid peroxidation [1,8,15,39,40]. It is accepted that both correlate to oxidative stress and cause the imbalance between the generation of oxygen derived radicals and the organism’s antioxidant potential [42,43]. CP-induced suppression of kidney antioxidant defense enzyme activity was also supported by the recently published experimental results [9,13,14].

The decrease in SOD activity (Figure 1) after CP administration might be due to the loss of copper and zinc which are essential for the activity of enzyme or to ROS induced inactivation of enzyme proteins [13,18,39]. CP has been demonstrated to induce the loss of copper and zinc in the kidneys. The decreased SOD activity is insufficient to scavenge the superoxoide anion, produced during the normal metabolic process [42,43]. The activities of CAT and GSH-Px (Figures 2 and 3) were also found to decrease after CP administration, resulting in the decreased ability of the kidney to scavenge toxic hydrogen peroxide (H_2_O_2_) and lipid peroxides. CP administration resulted in a decline of GSH concentration (Table 1) and a decrease of GR and GST activities (Figures 4 and 5) in the rat kidney. The results from the present study indicate that Se significantly reduces the depletion of GSH concentration and antioxidant defense enzyme activity in the kidney of rat treated with CP. The protective effects of Se seem to be primarily associated with its presence in the GSH-Px, which is known to protect DNA and other cellular components from damage by ROS. Selenoenzymes are also known to play roles in carcinogen metabolism, in the control of cell division, oxygen metabolism, detoxification processes, apoptosis induction, and the functioning of the immune system [19–21,24,25].

GSH is necessary for resistance to oxidative stress through detoxification of ROS. It can also detoxify many endogenous toxins, including CP, through the formation of GSH adducts [7,17,44,45]. In addition, the GSH redox cycle, which includes GSH, GSH-Px and GR, plays an important role in the detoxification of ROS that are generated by CP, so as to protect cells from the potential toxicity and carcinogenesis [17,18,44,46]. The primary symptoms of cisplatin nephrotoxicity are inhibition of protein synthesis and intracellular GSH and protein-SH depletion, resulting in lipid peroxidation and mitochondrial damage [2,10,44,45]. GSH and protein-SH form the major cellular antioxidant defense systems, which control lipid peroxidation. The reduced renal GSH can markedly increase the toxicity of CP. The depletion of GSH also seems to be a prime factor that permits lipid peroxidation in the CP-treated group. The treatment with Se was very effective in the prevention of oxidative damage induced by CP, which resulted in significantly increased GSH concentration [47]. Experimental studies demonstrated that exogenous GSH could offer protection against CP-induced renal injury. Thiols, such as the sulfur of GSH, bind to the platinum molecule, replacing one of the chloride ions, thus preventing binding to other cellular nucleophiles [45]. The increased intracellular GSH concentration correlated with decreased platinum-DNA binding in freshly isolated peripheral blood mononuclear cells [6]. Studies of tumor cell lines have shown a correlation between the increased levels of intracellular GSH and the resistance to CP [44,45].

GSH-Px, in particular, is highly dependent on GSH concentration. GSH-Px metabolizes H_2_O_2_ to water by using GSH as a hydrogen donor, resulting in the formation of GSSG [46,48]. GR subsequently regenerates GSH from GSSG. The decrease in the GSH-Px activity may result in the involvement of deleterious oxidative changes due to the accumulation of toxic products. The Se-containing enzyme GSH-Px protects cells against ROS. This result indicates that the increase in MDA in the kidney of rats treated with CP may be related to the decrease in the activity of GSH-Px. In this study, administration of Se exhibits GSH-Px-like activity, which prevented the decrease of GSH-Px activity in the kidney. Yoshida *et al.* [49] and Naziroğlu *et al.* [13] showed that the reduction of GSH-Px activity in the kidney tissue of rats treated with CP alone was prevented by co-treatment with Se.

The GST enzyme has an important role in detoxification of xenobiotics, drugs, and carcinogens, thus protecting the cells against redox cycling and oxidative stress. The decrease in the GST activity or –SH group could explain the induction of free radicals in CP-treated rats [16]. GST is a family of enzymes that catalyzes the conjugation of GSH to a variety of substrates. Several isoforms of GST have been shown to bind CP *in vivo* [44,50]. In the studies where increased resistance to CP was observed, none determined whether the inactivation of CP was due to GST binding to the CP, or catalyzing its conjugation to GSH. The previous studies show that after incubation of blood platelets with CP, the amount of GSH decreased and the complex of cisplatin with GSH (GS-cisPt) was formed (*via* reaction catalysed by GST) [50,51]. Feinfeld *et al.* [52] and Badary *et al.* [16] showed that the rats given a single toxic dose of CP excreted detectable GST activity in their urine, thus suggesting that urinary GST activity was a marker for a CP-induced proximal tubular damage.

The treatment with Se reversed the CP induced alterations of all examined parameters in rat kidney. The results indicate that Se significantly reduced the depletion of GSH concentration and antioxidant defense enzyme activity in the kidney of rats treated with CP, thus providing protection to the kidney. The protective effect of Se against CP-induced oxidative stress in this study could also be either direct, by inhibiting lipid peroxidation and scavenging free radicals [13,47], or indirect, through the enhancement of the activity of antioxidant defense enzymes including SOD, CAT and GSH-Px [9].

Se can reduce the nephrotoxicity and hepatotoxicity of CP without reducing the antitumor activity of the drug [13,20]. The protection correlates with higher levels of Se in the kidney and with higher concentration of GSH in the kidney, both compared to tumors. Se is known to protect GSH by forming the selenodiglutathione complex [24]. It is known that substitution reactions with biologic nucleophiles appear to govern the antitumor and toxic properties of platinum complexes [53]. Selenite is metabolized into selenols, specifically into methylselenol and glutathionylselenol, while bioactivation of selenite into selenols is a GSH-dependent process. HPLC with on line radioactivity detection of ^195m^Pt showed that methylselenol (HSe-CH_3_) was capable of forming a complex with CP *in vitro*. ^1^H-NMR gave evidence that the complex contained one or more Pt–Se–CH_3_ bonds. It is proposed that the formation of a CP-selenol complex also takes place *in vivo*, especially in the kidney, thereby preventing CP to exert its nephrotoxic activity [23,47]. The biological roles ascribed to Se include the prevention of cancer [19,54,55], cardiovascular disease and viral mutation [20–26,56].

## 5. Conclusions

In conclusion, we have shown that CP-induced nephrotoxicity is closely associated with the increase of lipid peroxidation in the kidney tissues. Treatment with CP causes significant changes in the activity of antioxidant defense enzymes (SOD, CAT, GSH-Px, GR and GST) and GSH concentration. The results of our study suggested that the treatment with Se reversed CP-induced lipid peroxidation and alterations in antioxidant defense system, and significantly prevented CP-induced kidneys damage. Our results showed that the nutritional antioxidant Se ameliorated oxidative stress and loss of cellular antioxidants and suggested that Se efficiently protected kidneys from CP-induced oxidative damage.

## Figures and Tables

**Table 1:** Effect of Se treatment on LPO and GSH concentrations in kidneys of CP-treated rats.

Parameters	Experimental groups			

	Control	CP	Se	Se + CP
LPO (nmol MDA/g tissue)	21.5 ± 2.8	27.3 ± 3.4 *	18.6 ± 1.5 **#**	23.7 ± 2.3 #
GSH (μmol/g protein)	28.9 ± 3.8	21.5 ± 3.1 *	26.7 ± 2.7 #	24.7 ± 2.7 #

Values are expressed as means ± SD; *n* = 6 for each treatment group; CP: Cisplatin; Se: selenium; LPO: lipid peroxidation; GSH: reduced glutathione;

* *p* < 0.05 compared with control group,

# *p* < 0.05 compared with CP group.
